# Importance of Metastatic Lesion Location and Subject‐Specificity in the Determination of Femoral Fracture Risk

**DOI:** 10.1002/cnm.70157

**Published:** 2026-03-16

**Authors:** Abbas Rizvi, Chloe E. H. Scott, Sohan Seth, A. Hamish R. W. Simpson, Pankaj Pankaj

**Affiliations:** ^1^ Institute for Bioengineering, School of Engineering The University of Edinburgh Edinburgh UK; ^2^ Edinburgh Orthopaedics, Royal Infirmary of Edinburgh Edinburgh UK; ^3^ Department of Mechanical Engineering Imperial College London London UK; ^4^ Institute of Machine Learning, School of Informatics The University of Edinburgh Edinburgh UK; ^5^ Department of Trauma and Orthopaedics, Edinburgh Medical School The University of Edinburgh Edinburgh UK; ^6^ Centre for Bone and Joint Health Queen Mary University of London London UK

**Keywords:** finite element modelling, fracture risk, metastatic bone disease, subject‐specificity

## Abstract

Evaluating the risk of pathological fracture in patients with bone metastasis continues to be a challenge. Clinicians use scoring systems such as Mirels', which are known to be unreliable. Patient‐specific finite element (FE) analyses have been shown to be more effective than empirical clinical guidelines. While patient‐specific FE models are valuable, they do not provide trends on fracture risk with respect to lesion locations that may apply across patients. Also, undertaking scans and conducting simulations for every patient is not practicable. Current knowledge is limited regarding the effect of lesion location in the femur and influence of patient‐specific factors on fracture risk. We developed an automated system that generates synthetic spherical lesions of uniform size, systematically shifts their location by 1 mm, and evaluates the resulting effects on the mechanical response to loading applied at the femoral head aligned to the mechanical axis. To evaluate the importance of subject‐specificity, models developed from CT scans of three cadaveric femurs and a generic Sawbones model were analysed for their mechanical behaviour for similar variation in lesion location. We found that lesion location plays an extremely important role in the determination of fracture risk, and that the trends associated with location are similar across subjects. Lesions in the femoral diaphysis with no cortical involvement have no distinguishable impact, while loss of cortical bone and their location from medial to lateral and along the shaft (proximal‐, mid‐ and distal‐diaphysis) have a key role in predicting potential fracture. We also found that normalised loss of stiffness when the lesion is on the medial side is almost two times that on the lateral side, as long as there is some cortical involvement in the proximal‐ and mid‐diaphysis.

## Graphical abstract

We investigated how lesion location and subject specificity affect the femur's mechanical response in metastatic bone disease. Using one generic and three subject‐specific models, we found that lesion location is a major determinant of fracture risk, with consistent trends across subjects.

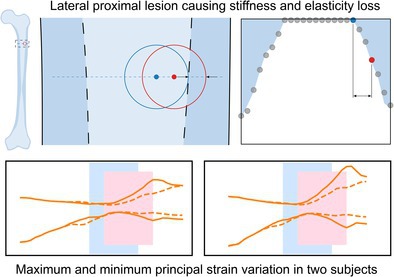

## 1 Introduction

Metastasis is the spread of primary cancer in advanced disease to distant organs such as the lungs, liver, bone or brain. Cancer originating in the breast, prostate, thyroid, kidney or lung [1] may spread to bones, resulting in metastatic bone disease (MBD). MBD causes abnormal tissue formation in bones, referred to as lesions, which cause pain on weight‐bearing, reduced load‐bearing capacity and increased fracture risk. Fractures significantly impact quality of life and increase mortality rates. Cases with risk of fracture require urgent assessment and consideration of surgical management by orthopaedic surgeons, as timely surgery can improve outcomes.

Medical professionals may perform prophylactic fixation, but this can lead to complications [2]. Conducting a risk assessment is important for making informed intervention decisions [3]. The scoring system by Mirels [4], based on the anatomical location of the lesion, its size, nature and pain severity, is part of the guidelines endorsed by the British Association of Surgical Oncology [5] to help clinicians in their decisions. A criterion by Harrington [6] which precedes Mirels' score considers cortical bone destruction for risk classification. These methods are easy to apply but have low sensitivity, making the decision unreliable [7]. Given the difficulty in accurately predicting fracture risk, the best results are believed to come from detailed, patient‐specific simulations based on biomechanics [8, 9, 10]. These methods, which use models from patient‐specific CT scans [11], require ethical permissions, human resources and software licences. Also, these models depend on bone density evaluation, which varies with the scanner [12], and its conversion to mechanical properties.

Most FE studies have been based on individual patient models, which limits the number of cases to a few commonly occurring lesion locations. Often studies use disease‐free cadaveric bones with trans‐cortical surgical defects created through material removal [13]. Synthetic spherical defects have also been made virtually in FE models [14, 15]. For example, Sas et al. [16] created synthetic lesions in scanned cadaver models to predict failure loads. This study was later reproduced by Gardegaront et al. [17] and showed that failure loads are highly sensitive to bone density variations. Amendola et al. [18] considered the effect of lesion location by creating lesions that penetrated the entire cortex, which is often regarded as a pathological fracture. Keyak et al. [19] also discussed the sensitivity of the location by moving the lesion inwards, but did not take into account the cortical involvement.

In this study, we developed an automated system that generates synthetic spherical lesions, of uniform size, and systematically shifts their location by 1 mm, with the aim to evaluate the sensitivity of lesion location on the mechanical response of the femur. This study employs FE modelling of both subject‐specific cadaver specimens and a standardised Sawbones (Pacific Research Laboratories Inc., Vashon, USA) model to identify patient‐independent trends that can guide clinical decision‐making for bones compromised by MBD.

## 2 Methods

Subject models were created using scans from three different defrosted cadaveric femurs (Table 1). Donor age, sex and body weight were not made available to the authors. Each femur was disarticulated at the hip joint and was scanned at the Clinical Research Imaging Centre at the University of Edinburgh. The scans were obtained using Aquilion ONE CT (Toshiba Medical Systems Ltd., Crawley, UK), a helical, multislice CT scanner with a 35 row detector. The scans captured the legs with a resolution of 35 μm isotropic voxels with each slice measuring 0.5 mm. The tube voltage and current were 225 kV and 300 mA, respectively, with a rotation time of 750 ms. A standard reconstruction was used, and the scans were exported as DICOM files (standard format for medical imaging) containing Hounsfield unit (HU) as greyscale values, details shown in Table 2. Each model was calibrated using hydroxyapatite CaHA phantoms (Image Analysis Inc., Columbia, USA) with known densities of 0, 75 and 150 mg/cm^3^. These densities are lower than the typical apparent densities of cortical bone; however, the approach is consistent with previous CT‐based FE studies [20, 21] that extrapolate the linear HU–density relationship beyond the range of the phantoms. The femurs were manually segmented from the scans using commercially available software ScanIP (Synopsys Inc., Sunnyvale, USA).

**TABLE 1:** Comparison of different femur models.

	Subject			Sawbones
	1	2	3	
Left/right	R	L	L	L
Femoral head diameter (mm)	51	50	52	52
Neck‐shaft angle (°)	125	121	127	113
Femoral anteversion (°)	10	2	19	0
Femoral shaft length (mm)	382	404	371	410
Intercondylar width (mm)	91	84	88	90
Diaphyseal diameter (mm)	35	35	35	36

**TABLE 2:** Calibration of different cadaver models.

	Resolution (mm)		HU (phantoms)			ρ=a+b×HU		
	In‐plane	Interval	0	75	150	*a* (10^−5^)	*b* (10^−7^)	*R* ^2^
Subject 1	0.770	0.25	25	125	245	1453	6799	0.997
Subject 2	0.728	0.25	10	115	225	6381	6975	0.999
Subject 3	0.587	0.25	10	115	235	4882	6657	0.999

Abbreviation: HU, Hounsfield unit.

Bone was assumed to be linear‐isotropic and elastic, but heterogenous, as in many previous studies [22]. Phantom densities were used to calculate bone density in the scans $\rho_{\mathrm{QCT}}$ (in g cm^−3^) through regression, using $\rho_{\mathrm{QCT}}=a+b\times \mathrm{HU}$ where the calculated values for a and b are shown in Table 2.

**FIGURE 1:**
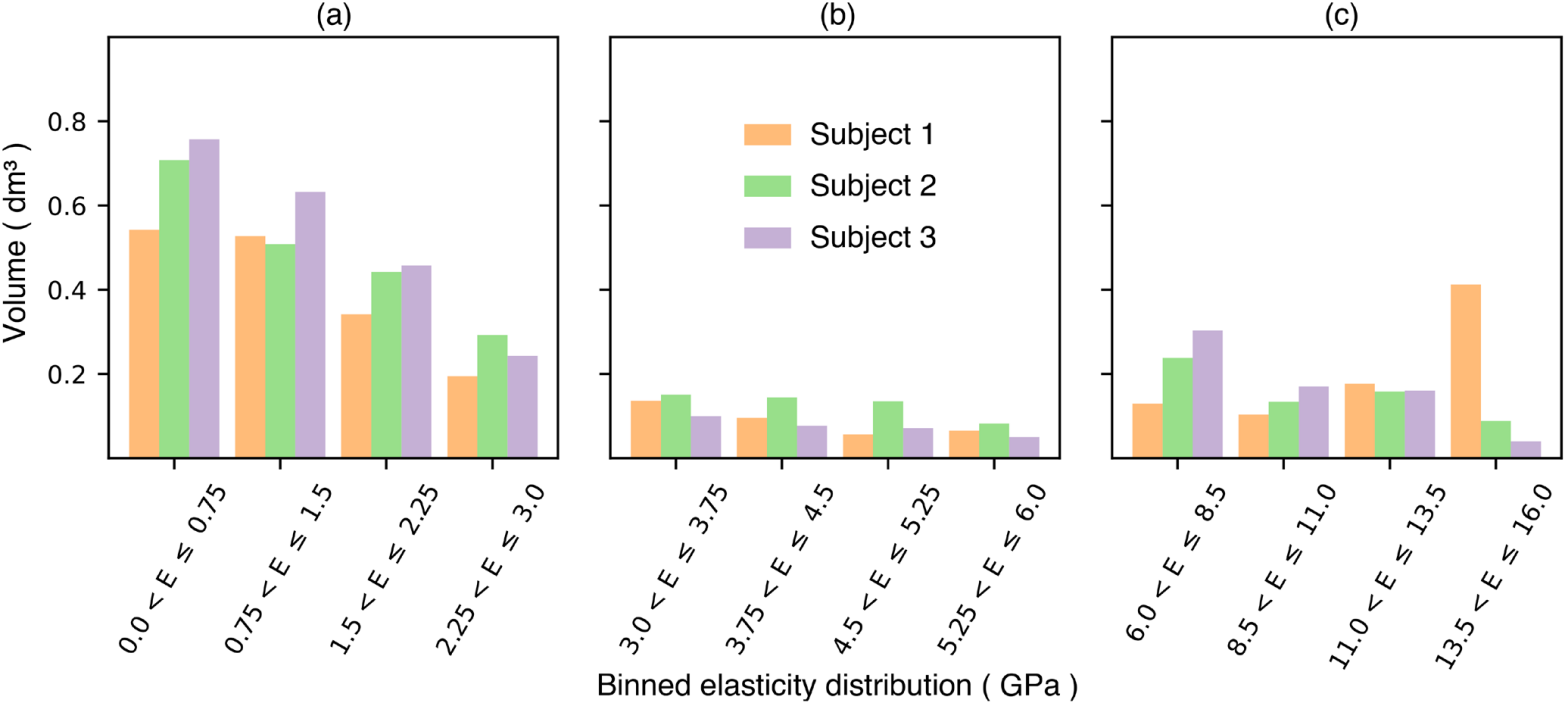
Distribution of the modulus of elasticity in the three cadaver femurs. For each femur, the histograms represent the entire bone volume, sub‐grouped into trabecular region (a); mid‐range moduli region (b); and cortical region (c).

The $\rho_{\mathrm{QCT}}$ was first converted to ash density $\rho_{\mathrm{ash}}$ using $\rho_{\mathrm{ash}}=0.0633+0.877\times \rho_{\mathrm{QCT}}$ for CaHA, and then apparent density $\rho_{\mathrm{app}}$ using $\rho_{\mathrm{app}}=\rho_{\mathrm{ash}}/0.626$. The Young's modulus in MPa was computed as $E=6850\rho^{1.49}$ [23]. The HU values were partitioned into 100 sets, resulting in 70–100 valid material properties, which were assumed to represent a reasonable variation. The corresponding elasticity distribution for the three cadaver models is shown in Figure 1. A Poisson's ratio of 0.3 was used for all materials [24].

For comparison, a standardised model of a fourth‐generation Sawbones femur was also modelled. The Sawbones model features distinct cortical and trabecular regions (notable in the proximal and distal ends). Young's modulus of 16.7 GPa for the cortical bone and 155 MPa for the trabecular bone were assigned to the Sawbones model [25]. After segmentation, each model was exported as a linear tetrahedral mesh for analysis.

Since the Sawbones model has a well‐defined cortical region, it provides an easy way to study the effect of cortical destruction caused by a lesion. The volume of cortical destruction was evaluated as the total volume of elements in the cortical region that have transformed into a lesion. The penetration of the lesion from the edge of the cortical‐trabecular boundary into the cortical region was termed transverse cortical involvement (TCI).

The location of the hip joint centre was calculated by fitting the nodes of the femoral head surface to a sphere using the least‐squares method. The hip joint centre was used as the reference origin of the global coordinate system for all models [26]. The neck axis was defined as the line passing through the fitted centre of the femoral neck and the hip joint centre. The anatomical shaft axis was determined as the line passing through centres, calculated through surface fitting at the proximal and distal diaphysis. The femur models were oriented such that the anatomical shaft axis was parallel to the sagittal axis, and the projection of the posterior condylar line was parallel to the coronal axis. The knee joint centre was set at the midpoint on the epicondylar line, and the mechanical axis was defined as the line passing through the hip joint centre and knee joint centre. The geometrical details of different models are shown in Table 1. After the registration, all meshes were reoriented for consistency.

In the FE model, all nodes on the femoral head were tied to a single reference point positioned at the hip joint centre. The nodes on each distal condyle were tied to separate reference points located at the fitted centre of the respective condyle, and both distal reference points were fully constrained in all degrees of freedom. A load was then applied to the femoral head, aligned with the mechanical axis, to emulate single‐legged stance. This loading condition is similar to ones commonly used in the literature [10], although some studies explicitly include muscle forces and consider additional physiological activities [27, 28].

In order to maintain consistency amongst subjects, the knee joint centre was adjusted such that a reaction force at the medial condyle was 55% of the total, and the direction of femoral head displacement was in the coronal plane. The 55:45 ratio between the reaction forces on the medial and lateral distal condyles represents the average force distribution during the midstance phase [29]. This ensured similarity of loading across subjects. No restriction was applied to the hip joint centre; therefore, its deflection in the coronal plane was also consistent for all models considered.

To ensure the accuracy and reliability of the FE model, a mesh convergence study was conducted on disease‐free femurs. Multiple meshes with varying densities were considered, with the number of elements varying from around 300,000 to 2,000,000. The displacement of the femoral head for the loading considered was around 3% higher for a mesh with approximately 1,500,000 elements in comparison to the coarsest mesh. Further increase to 2,000,000 elements resulted in a displacement increase of 0.3%. So, meshes with approximately 1,500,000 elements were adopted for subsequent analyses. The element volumes followed a normal distribution, with a mean of 0.4 mm^3^ and a standard deviation of 0.2 mm^3^. Only around 1.5% of elements had edge lengths greater than 2.5 mm.

In order to demonstrate the impact of a lesion on fracture risk, a large load, representing 7.5 times the average body weight (approximately 68 kg), was applied. This approach aligns with previous studies, which have considered a diseased femur capable of withstanding a load in excess of 7.5 times the body weight to be at low risk of fracture [30].

Failure initiation in bone is known to depend on principal strains [31], with distinct thresholds for tension and compression [32]. A reduction in stiffness has been shown to indicate a corresponding decrease in strength [33]. Therefore, both strain and stiffness were used as indicators of failure susceptibility for different lesion locations. Strain‐based plasticity algorithms have been previously proposed for bone [34, 35] to simulate post‐elastic behaviour. Some other previous studies have used the Hoffman criterion [18, 27], which is essentially a stress‐based criterion to define post‐elastic behaviour of bone. In this study, we employed simple strain‐based elasticity to evaluate maximum and minimum principal strains in our region of interest to evaluate possible risk of fracture as done in a population‐based finite element study conducted by Taddei et al. [36].

Metastatic lesions were simulated by reducing the Young's modulus of elements within a spherical region of the femoral mesh to 50 kPa. In Abaqus, this was implemented by defining material properties as field‐variable dependent and then modifying the field values assigned to the lesion elements. The outcomes obtained with this procedure were consistent with those from lesions created directly in the CT data.

**FIGURE 2:**
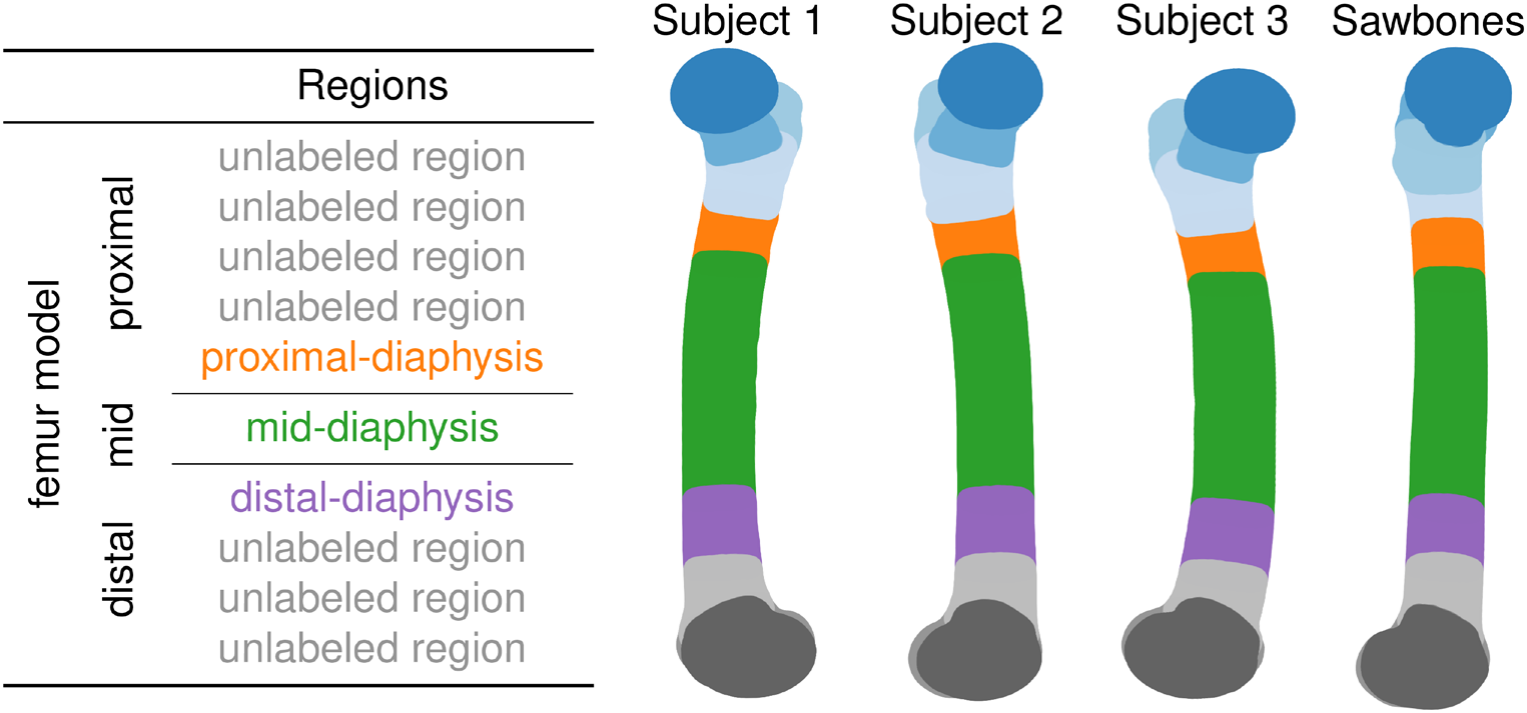
Division of femur model into multiple regions using k‐means clustering.

Automation of this procedure enabled the creation and repositioning of lesions with desired diameters at multiple locations within the femur. Three regions along the femoral shaft were considered. To reduce selection bias, and enhance reproducibility across subjects, the locations used were determined using k‐means clustering with a fixed random seed. The nodes of each model were divided into regions and then subregions, as shown in Figure 2. The centroids of the proximal‐, mid‐ and distal‐diaphysis regions were used to define the transverse‐plane locations. Lesion centres were shifted in 1 mm increments between the medial and lateral sides of the shaft, following a path aligned with the shaft axis. A lesion diameter of 10 mm was selected as a nominal size, large enough to ensure consistent representation within the FE mesh for all subjects regardless of lesion location, yet sufficiently small to avoid interference with adjacent anterior and posterior regions, which would have otherwise interfered with the similarity of lesions in different subjects. For the four models, a total of over 300 cases were considered.

As subject‐specific models do not have a well‐defined cortical‐trabecular interface, a different approach was considered for these cases. We used weighted average elasticity‐loss due to the presence of lesion to represent cortical volume destruction. The elasticity‐loss was evaluated as

(1) $$\text{elasticity‐loss}=\frac{\sum_{i=1}^{n}V_{i}E_{i}^{\left(1\right)}-\sum_{i=1}^{n}V_{i}E_{i}^{\left(0\right)}}{\sum_{i=1}^{n}V_{i}}$$

where n is the number of elements in the lesion, and $V_{i}$, $E_{i}^{\left(1\right)}$ and $E_{i}^{\left(0\right)}$ represent the volume, elasticity with the lesion, and elasticity without the lesion of element i, respectively.

**FIGURE 3:**
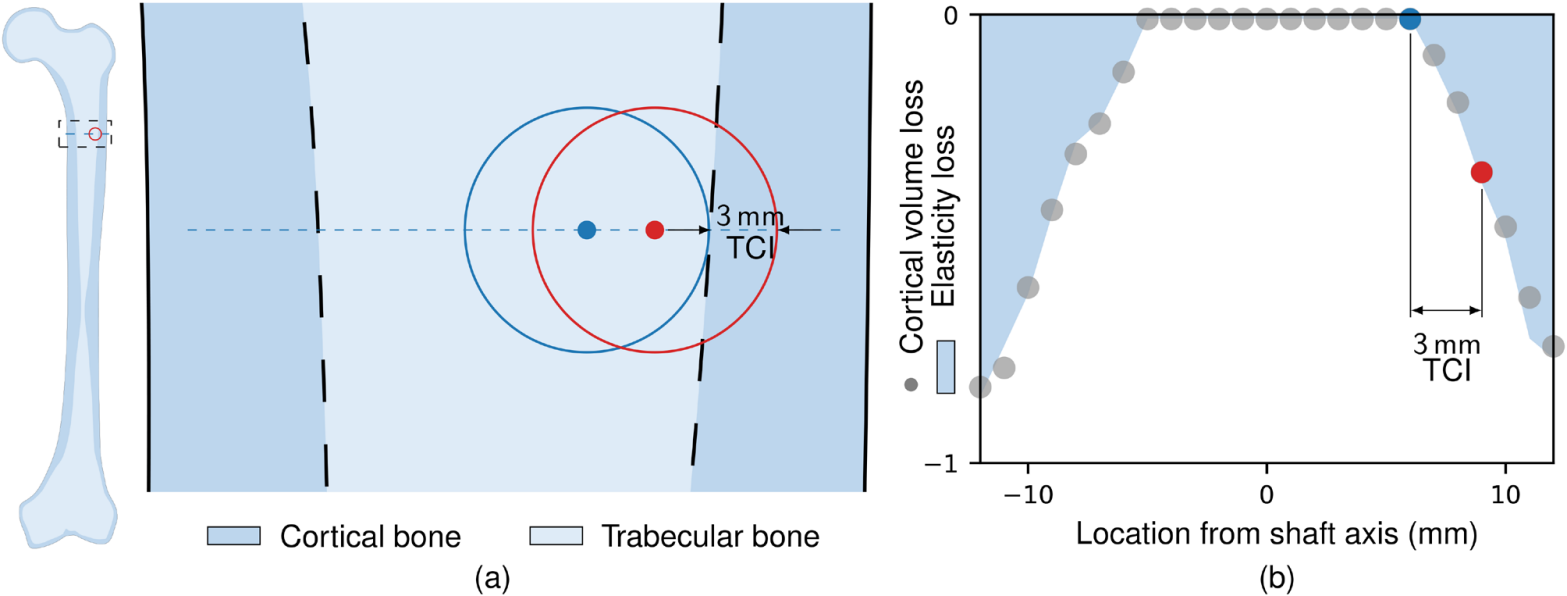
The Sawbones model (a) features a magnified section showing trabecular and cortical bone. Lesion location varies along the dashed line from medial to lateral. The red and blue circles represent 10 mm lesions with their centres represented by dots. (b) Bone destruction for the Sawbones model, with cortical volume loss (dots) and elasticity‐loss (shaded dark) as the lesion location shifts from medial to lateral. The red highlights a case with 3 mm of TCI on the lateral side.

Figure 3 illustrates the simple link between elasticity‐loss and cortical volume loss for a 10 mm lesion as it moves from the medial to the lateral side for the Sawbones model. This and subsequent images are oriented such that the medial side is on the left. The locations in the diagrams shown along the medial‐lateral in transverse plane are all defined with respect to the shaft axis. The similarity between the normalised volume of cortical bone loss in the Sawbones model and elasticity‐loss is apparent as the lesion traverses.

The stiffness of the femur designated by the load required to displace the femoral head by unit displacement was one of the criteria used to evaluate the effect of varying lesion location. Increase in flexibility or reduction in stiffness was an indicator of reduced strength. The stiffness‐loss was evaluated as:

(2) $$\text{stiffness‐loss}=\frac{R^{\left(1\right)}}{U^{\left(1\right)}}-\frac{R^{\left(0\right)}}{U^{\left(0\right)}}$$

where $R^{\left(1\right)}$ and $U^{\left(1\right)}$ are the magnitudes of applied force and displacement of hip joint centre, respectively, for a case with a lesion, while $R^{\left(0\right)}$ and $U^{\left(0\right)}$ are the corresponding force and displacement of hip joint centre for the lesion‐free model.

## 3 Results

**FIGURE 4:**
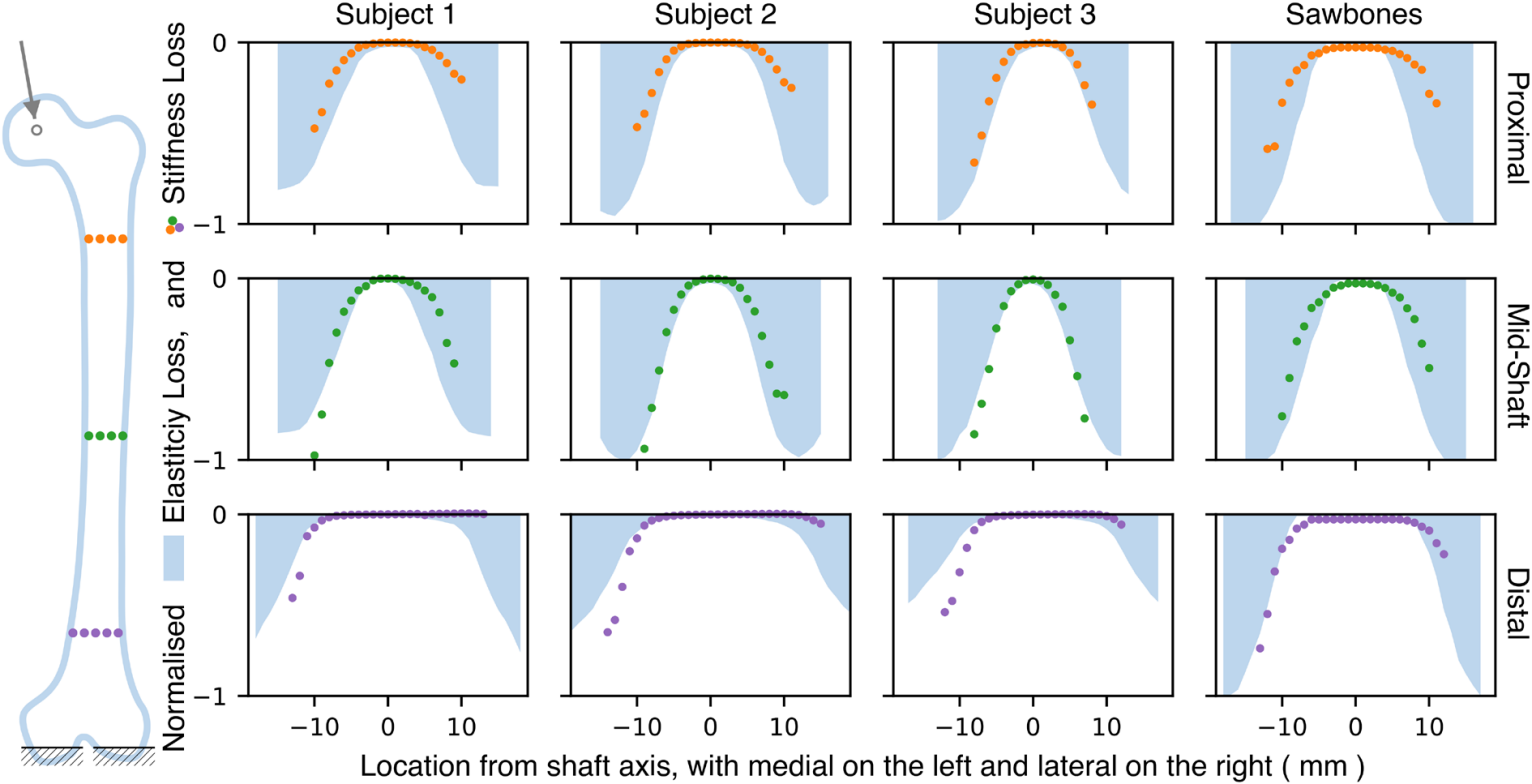
Sensitivity analysis for stiffness‐ and elasticity‐loss for 10 mm lesions at varying locations, across three subjects and the Sawbones model.

Stiffness‐loss was evaluated in the three selected regions along the femoral shaft. Figure 4 illustrates the impact on stiffness‐ and elasticity‐loss, as the location of a 10 mm lesion varies from medial to lateral locations (shown on the *x*‐axis), at the three positions along the femoral shaft. The stiffness‐loss has been normalised with respect to the largest stiffness‐loss value for each femur; the corresponding maxima and lesion locations are reported in Table 3. Although the quantitative magnitude of stiffness‐loss varies between subjects, all subjects show a similar trend for normalised elasticity‐loss, with higher and symmetrical magnitudes at the medial and lateral ends. The distal regions have a larger cross‐section and reduced elasticity‐loss. The Sawbones model is an exception, as it maintains cortical thickness along the shaft. The stiffness‐loss plot only displays cases in which the lesion has not extended beyond the bone, whereas the average elasticity‐loss plot is confined to the edge of the bone.

**TABLE 3:** Comparison of the maximum values and their corresponding locations across all cases with a 10 mm lesion, evaluated among different femur models.

	Subject			Sawbones
	1	2	3	
Elasticity‐loss (GPa)	−18.0	−11.1	−11.8	−16.7
Stifness‐loss (kN/m)	−29.4	−17.7	−11.8	−25.2
Location along shaft	Mid	Mid	Mid	Mid
Location along plane	Med	Med	Med	Med

Abbreviations: Med, medial; Mid, mid‐shaft.

For all subjects, the magnitude of stiffness‐loss increases as the lesion location moves from the proximal to the mid‐shaft and then decreases when the lesion is in the distal region. The greatest stiffness‐loss is observed at the mid‐shaft. When comparing lesions on the medial side of the proximal and distal shaft locations, the stiffness‐loss is similar at both locations, even though the elasticity‐loss is lower in the distal region. The Sawbones model, which has higher cortical elasticity, shows greater stiffness‐loss. There is an apparent similarity between elasticity‐ and stiffness‐loss in all cases, except when the lesion is on the lateral side, and particularly at the distal end. The elasticity‐loss on both medial and lateral sides is similar, but the presence of the lesion on the medial side has a greater impact on stiffness‐loss.

**FIGURE 5:**
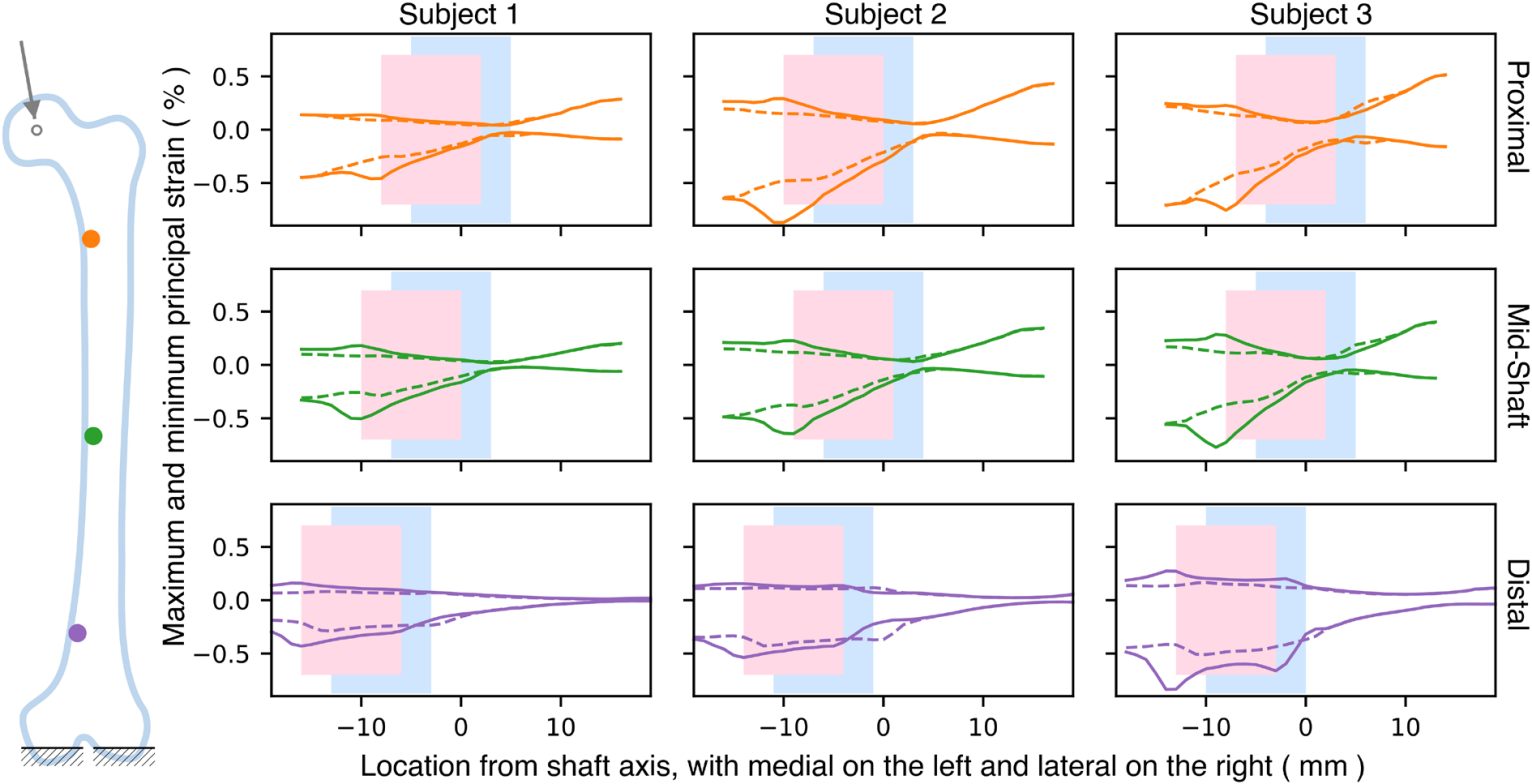
Maximum and minimum principal strain variation for cases with a 10 mm lesion having 0 and 3 mm TCI on the medial side across the three subjects. Blue and pink boxes indicate lesion locations for 0 and 3 mm of TCI, respectively, with a dashed line representing 0 mm and a solid line representing 3 mm.

**FIGURE 6:**
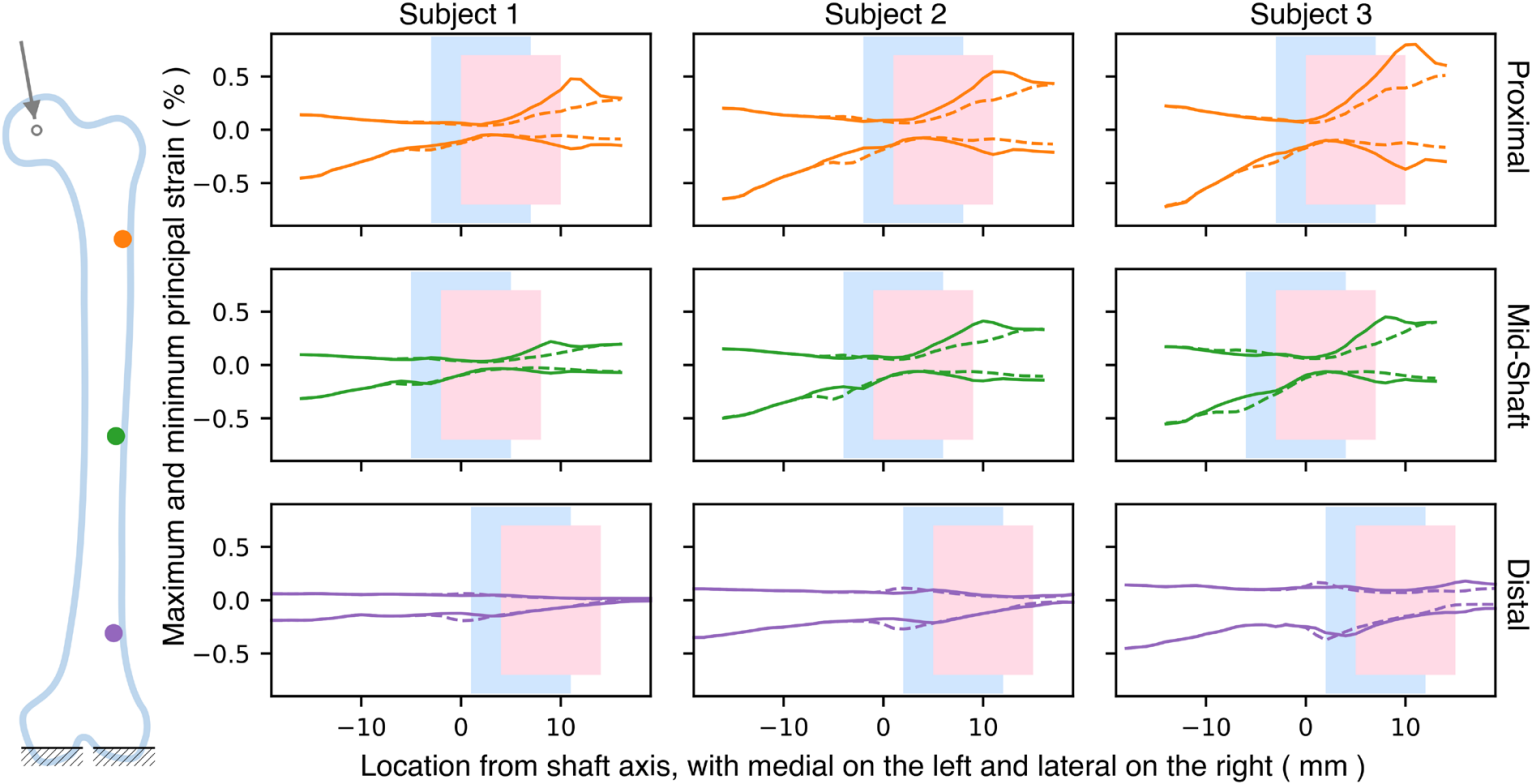
Maximum and minimum principal strain variation for cases with a 10 mm lesion having 0 and 3 mm TCI on the lateral side across the three subjects. Blue and pink boxes indicate lesion locations for 0 and 3 mm of TCI, respectively, with a dashed line representing 0 mm and a solid line representing 3 mm.

To examine the effect of lesions on strain, selected cases with lesions on the medial and lateral sides were compared (Figures 5 and 6). Figure 5 shows the maximum (tensile) and minimum (compressive) principal strain variation along the line perpendicular to the shaft axis for cases with a 10 mm lesion, having approximately 0 and 3 mm TCI on the medial side for the three subjects. To prevent issues associated with strain concentrations due to element size and heterogeneous properties, the strains were calculated by taking the mean of a spherical region of 1.5 mm radius around the observed point.

All cases across all subjects show similar trends, except for Subject 3 with the lesion on the distal side. By examining the series of cases spaced at 1 mm intervals, we identified the case with a TCI of 3 mm by selecting the third instance that exhibited an increase in elasticity‐loss when the lesion moved from the trabecular to cortical region. Subject 3 has a small cortical thickness and 3 mm TCI results in significant cortical destruction. For all subjects, the peak magnitude of minimum principal strains is higher than that of maximum principal strains. The magnitudes are smaller for the mid‐shaft region in comparison to the proximal region. Similarly, Figure 6 shows maximum and minimum principal strains when the 10 mm lesion with 0 and 3 mm of TCI is on the lateral side. The overall magnitudes of strain are lower than those of Figure 5, when the lesion is on the medial side. For all subjects, the peak magnitude of minimum principal strains is found to be higher than that of maximum principal strains.

## 4 Discussion

This study aimed to explore the impact of lesion location and the importance of subject‐specificity. Analyses using 10 mm lesions examined how changes in location affect stiffness‐loss and principal strains. This study shows that lesion location plays an extremely important role in the determination of fracture risk using the indicators considered, and the trends associated with location are similar across subjects. Lesions confined within the trabecular region have no distinguishable impact, indicating that femur fracture prediction depends mainly on cortical loss and its location.

We employed elasticity‐loss, as a proxy for cortical bone loss. There is an apparent relationship between elasticity‐loss and cortical destruction caused by a lesion. The trabecular region's elastic modulus is typically two orders of magnitude lower than that for the cortical region, so elasticity‐loss is an effective representation of cortical volume loss. This approach is simple to apply in subject‐specific models, which have inhomogeneous material properties and ill‐defined cortical trabecular boundary. Defining regional boundaries and describing volume destruction is challenging [37], and has been only possible with high resolution 3D scans [38].

The automated process used in this study for the creation of synthetic lesions in FE models permitted systematic variation of their location and evaluation of their effect across subjects. This approach showed that elasticity‐loss displayed similar trends across the three subjects considered. The trend for normalised stiffness‐loss was very similar across subjects. Stiffness‐loss in the femoral diaphysis is only observable with cortical involvement. The thick cortical bone at the diaphysis means that its involvement in a lesion results in significant elasticity‐loss. The largest stiffness‐loss is seen when the lesion is in the mid‐shaft, and on the medial side. Our analysis showed that the stiffness‐loss was most affected by the volume of cortical destruction. In assessing femur fracture risk, clinicians often prioritise cortical destruction as a key parameter [8].

For the loading considered, the femoral shaft is subjected to bending towards the medial side along with axial compression. Therefore, the medial side experiences combined axial and bending compression, while the lateral side experiences combined axial compression and bending tension. This is reflected in reduced stiffness‐loss when lesions are on the lateral side, and is most apparent when they are on the distal end. This is also apparent from principal strains; the minimum principal (compressive) strains increase significantly (in comparison to those in a lesion‐free femur) when the lesions are on the medial side. When lesions move to the lateral side, where a combination of tension and compression exists, there is only a slight increase in maximum principal strains, particularly in the distal and mid‐shaft regions. Previous studies on lesion‐free femurs have also shown that principal strain values are typically higher on the medial side than the lateral side, decreasing from proximal to distal regions under walking and stair‐climbing loads [39]. This needs to be considered in the context of bone's asymmetry in yield strain, which is larger in compression than in tension [32], and can be incorporated in non‐linear computational analysis [34]. Studies with case‐specific statistical analyses using FE have similarly indicated that medial cortical thinning has a higher risk than equivalent thinning on the lateral, anterior or posterior sides [40].

The observed pattern, where the magnitude of the stiffness‐loss initially increases and then decreases as lesions move to distal regions, suggests the influence of two interacting factors. The first is the lesion's distance from the mechanical axis, which also regulates the patterns of principal strains described above. The second factor is the elasticity‐loss, which tends to decrease in the distal region for subject‐specific models.

In this study, the synthetic lesions were designed to be relatively small and spherical. This choice enabled a targeted assessment of their impact on specific regions. Spheres were preferred over randomly orientated ellipsoids [41] because they offered a consistent approximation of lesion shape. Spheres closely mimic the appearance of smaller lesions seen in medical images of patients with MBD. As the mesh employed tetrahedral elements, the sphere representing lesions had a slightly jagged circumference. These jagged edges did not affect the results, which is evident from the smoothness of stiffness‐loss plots obtained as lesions traversed through the bone.

This study focused on a specific loading condition, which is the mid‐stance phase of the gait cycle. This phase resembles a single‐legged stance, where most of the body weight is supported by one leg, and has been extensively used in computational and in vitro studies [11, 15, 42, 43]. Therefore, this loading case is an appropriate choice for evaluating trends associated with the effect of lesion location. Our results, such as those showing that principal strain values are typically higher on the medial side than the lateral side, have been shown to exist under walking and stair‐climbing loads [39] for disease‐free femurs. However, for more comprehensive results, future research could consider other loading conditions.

Currently, there are no existing measurement techniques capable of evaluating internal stresses and strains within the femur. Lesions that have been created for in vitro tests using machining (from the exterior) and surgical tools are only case‐specific [8, 14, 17]. While such approaches are useful for testing numerical models, they do not replicate a MBD scenario. Individual case studies that use numerical models to evaluate fracture risk by examining localised stresses and strains are highly dependent on processes used, for example, scanning method and property assignment. Eggermont et al. [12] showed that case‐specific FE analyses heavily rely on the process of determining the material properties of scanned bones. Therefore, the present study focuses on trends rather than on specific values. Our analyses show that while lesion‐induced strains can inform failure risk, much of this risk can be inferred based simply on the location under consideration. This is consistent across the models considered. As stated earlier, we used principal elastic strains and stiffness‐loss to evaluate fracture risk. Similar approaches have been previously used [33, 36] to indicate fracture initiation. Some previous studies, however, have employed nonlinear models. For example, Goodheart et al. [27] and Amendola et al. [18] used the Hoffman criterion to model post‐elastic behaviour, though this criterion is essentially stress‐based and for anisotropic material modelling.

By investigating the impact of small lesions, we were able to assess the relative sensitivity of different locations. However, this approach limited our ability to examine absolute measures of risk. In contrast, other studies such as Damron et al. [44], that use larger lesions, have been able to define specific values of structural rigidity loss as indicative of high risk. The study was limited to application of a simplified single joint load to maintain consistency amongst subjects while maintaining 55:45 (±0.1) ratio for reaction forces on distal condyles as reported by previous studies [29]. Some previous studies such as [45] have considered the effect of a range of muscle forces and showed that inclusion of abductors can influence response, particularly in the proximal region. Another study on femurs with MBD has shown that exclusion of physiological loads resulted in higher strains and lower strength [28]. Our analysis was limited to lesions located within the femoral diaphysis. Metastatic lesions, however, are also common in the intertrochanteric and femoral neck regions. In these regions, the geometry, load transfer and cortical density differ substantially from those in the shaft. Therefore, additional criteria may be required to evaluate fracture risk.

In conclusion, our study shows that lesion location plays a key role in the determination of fracture risk, and the risk trends are largely independent of subject specificity.

## Funding

This research was supported by Higher Education Commission, Pakistan, which provided financial support to the first author. This support is gratefully acknowledged.

## Ethics Statement

This statement relates to the subject‐specific CT scans used in this study. This investigation took place at the Department of Anatomy, University of Edinburgh, and was carried out in accordance with the Human Tissue (Scotland) Act 2006. The imaging was conducted under the supervision of a licenced anatomist at the Clinical Research Imaging Centre, Queen's Medical Research Institute, Royal Infirmary of Edinburgh, University of Edinburgh.

## Conflicts of Interest

Pankaj Pankaj is an Associate Editor for the *International Journal for Numerical Methods in Biomedical Engineering* (*IJNMBE*) journal. The other authors declare no conflicts of interest.

## Data Availability

The data that support the findings of this study are available on request from the corresponding author. The data are not publicly available due to privacy or ethical restrictions.
